# Full Parametric Study of the Influence of Ionomer Content, Catalyst Loading and Catalyst Type on Oxygen and Ion Transport in PEM Fuel Cell Catalyst Layers

**DOI:** 10.3390/molecules25071523

**Published:** 2020-03-27

**Authors:** Robert Alink, Rajveer Singh, Patrick Schneider, Kläre Christmann, Johannes Schall, Roman Keding, Nada Zamel

**Affiliations:** Fraunhofer Institute for Solar Energy Systems, ISE, Heidenhofstrasse 2, 79110 Freiburg, GermanyRajveer.Singh@ise.fraunhofer.de (R.S.); patrick.david.schneider@ise.fraunhofer.de (P.S.); klaere.christmann@ise.fraunhofer.de (K.C.); johannes.schall@ise.fraunhofer.de (J.S.); roman.keding@ise.fraunhofer.de (R.K.)

**Keywords:** cathode catalyst layer, I/C ratio, diffusion limitation, conductivity limitation

## Abstract

To advance the technology of polymer electrolyte membrane fuel cells, material development is at the forefront of research. This is especially true for membrane electrode assembly, where the structuring of its various layers has proven to be directly linked to performance increase. In this study, we investigate the influence of the various ingredients in the cathode catalyst layer, such as ionomer content, catalyst loading and catalyst type, on the oxygen and ion transport using a full parametric analysis. Using two types of catalysts, 40 wt.% Pt/C and 60 wt.% Pt/C with high surface area carbon, the ionomer/carbon content was varied between 0.29–1.67, while varying the Pt loading in the range of 0.05–0.8 mg cm^−2^. The optimum ionomer content was found to be dependent on the operating point and condition, as well as catalyst loading and type. The data set provided in this work gives a starting point to further understanding of structured catalyst layers.

## 1. Introduction

Further advancement of polymer electrolyte membrane (PEM) fuel cells, particularly for use in the automotive industry, must be achieved as a balance between cost and functionality. The catalyst layer as the heart of the cell controls the half-cell reactions and their products. Its structure governs the various transport phenomena simultaneously taking place and affects its overall activity, stability and life time. Throughout the years, the optimization of the structure of the catalyst layer, with special attention given to the cathode, has been achieved via systematic optimization of its components [1]. The importance of this optimization stems from the heterogeneous and complex structure of the layer, which must fulfill its main four requirements: (1) existence of a three-phase interface on which the reaction occurs, (2) continuous path for efficient transport of protons, (3) a continuous pore network for the transport of reactants/products and for efficient water removal, and (4) continuous passage for the conduction of electrons between the catalyst layer and the current collector. Efforts to increase the stability and durability of the catalyst layer are hence ongoing with one such effort focusing on the differences in structure between conventional and non-conventional catalyst layers [1]. Examples of non-conventional catalyst layers are dispersed catalyst layers, either on the membrane or the gas diffusion layer, ultrathin catalyst layers and nano-structured thin film (NSTF) catalyst layers [2,3,4,5,6]. Although much work is found on non-conventional catalyst layers, conventional layers are still at the forefront of research due to other challenges facing their non-conventional counterparts [1] and hence, the discussion within this manuscript is focused on conventional catalyst layers.

The structure of conventional catalyst layers specifically is shaped by the ink, which is composed of a catalyst deposited on a support (e.g., Pt/C), ionomer and a dispersing solvent. The dispersion medium governs the ink properties, which ultimately govern the physical and mass transport properties of the catalyst layer. In [7], a thorough review, focused on the analysis of the colloidal ink, was put forward, where the authors highlighted the importance of understanding the ink properties from a nanoscale in order to understand the macroscale effects. Hence, understanding the interaction between the layer’s ingredients, its structure and performance is important to the advancement of PEM fuel cells. Another way to optimize the layer performance is to introduce gradients to adjust to inhomogeneous conditions that occur during fuel cell performance. These gradients can occur in all three dimensions, including the in-plane (x-y) and through-plane (z) dimensions of the catalyst layer.

The effect of catalyst layer structuring on the performance of PEM fuel cells was investigated by various groups in literature. This was mainly carried out by the effect of graded layers taking into consideration specific ingredients. Chen et al. [8] investigated the performance of a cell manufactured with cathode catalyst layers having two layers of different Pt/C ratio and Nafion content. They showed that the region where the reaction occurs can significantly affect the performance of the cell. Allocating more Nafion and Pt/C in the sublayer closer to the membrane was shown to significantly improve the performance. This finding, however, does not agree with the experimental study by Yoon et al. [9]. In [9], the authors prepared multi-layer structured cathodes by spray-drying, where they varied the ionomer content in the thickness of the catalyst layer. In their study, they found that a structured catalyst layer does not affect the overall performance. To further understand the interaction between Pt and ionomer, a comprehensive numerical model of a single cell to investigate the spatial distribution of Pt loading and ionomer content in the through-plane direction was developed by Xing et al. [10]. They found that an optimal distribution is influenced by the voltage. They suggested that understanding these dependencies can ultimately help in the reduction of the Pt loading. Herden et al. [11], [12] investigated the effect of varying the ionomer equivalent weight in the in-plane direction on the performance of an automotive cell. The measurements were carried out on a segmented automotive cell, where the current density and temperature distributions were recorded. To do so, a membrane electrode assembly (MEA) with 772 and 825 g/mol ionomer equivalent weight was assembled in a segmented automotive cell, where the temperature and current density distributions were recorded. The performance of this MEA was then compared to two MEAs with homogeneous ionomer equivalent weights (one with 772 g mol^−1^ and another with 825 g mol^−1^). They showed that the structuring of the cathode catalyst layer with varying ionomer equivalent weight is important for water management within the cathode. Similarly, the through-plane variation of the ionomer equivalent weight was investigated by Shahgaldi et al. [13], [14] using in-house produced catalyst coated membranes (CCMs). In their work, they showed that the catalyst layer performance can be enhanced by the systematic design of the layer. By choosing the proper ionomer/Pt-gradient, the morphological and microstructural characteristics of the catalyst layer can lead to a reduced ionic resistance with improved mass transport capability, catalyst activity and Pt utilization. In another publication, the same authors [15] discussed the impact of the manufacturing process of the CCM, namely using the decal method, on the performance of the cell. The effect of the manufacturing process on catalyst coated membranes produced in the lab was also investigated in [16]. Sassin et al. [16] examined various production parameters using direct deposition of the catalyst layer on the membrane via ultrasonic spray deposition. They used this parametric analysis to investigate how production of the catalyst layer can ultimately affect the performance of the cell. In the work by Yu et al. [17], the use of reactive spray deposition would be beneficial in the production of low Pt loaded catalyst layers. They investigated the effect of I/C ratio in the catalyst layer on the performance of layers directly deposited on the membrane. Ex situ analyses, such as mercury porosimetry and Nitrogen adsorption, were used to investigate the pore distribution of the layers investigated. They found that regardless of Pt loading, the Brunauer–Emmett–Teller (BET) surface area and pore volume of the layers decreased with the increase in I/C ratio. They suggested that based on the results of their work, an enhanced performance at a low I/C ratio, the use of reactive spray deposition would be beneficial in the production of low Pt loaded catalyst layers.

Other works have focused on understanding the effect of a single ingredient on the performance of the cell. The use of short side chain (SSC) ionomer in the cathode catalyst layer was also shown to improve the performance of the cell in [18], especially at dry conditions. The benefit of using a SSC ionomer under dry conditions was shown to extend to operation at below zero temperatures [19]. In [19], the authors investigated the effect of side chain length on the durability of the catalyst layer subjected to freeze–thaw cycles between 30 and −40 °C They compared the degradation mechanism that occurs due to such temperature cycling and found that degradation in the presence of a long side chain ionomer is initiated by ionomer swelling and pore expansion, and then proceeds mainly through pore expansion. Whereas the degradation of catalyst layers with SSC ionomer was initiated by ionomer swelling and pore expansion, and proceeded through the detachment of large-scale CL flakes, and morphology and microstructure changes thereafter. Further, Shukla et al. [20] investigated cell performance, Tafel slope, reaction order and local oxygen transport resistance in order to obtain a relationship between Pt loading and performance. Pt loading is an important factor in determining the catalyst layer activity and hence, overall performance of the cell. 

Although much work has been carried out to investigate the performance of the cell dependent on the Pt loading or ionomer content/type, most of the work focuses on the variation of either parameter rather than the relationship between the various ingredients. This is also often carried out under a very limited range of variation. Hence, in this work, we produce a full parametric study of the three parameters: ionomer content (ultimately the ionomer to carbon ratio), platinum loading and Pt/C ratio (40 wt% and 60 wt% platinum on carbon). Using the decal transfer method, cathode catalyst coated membranes are produced in-house with Pt loadings from 0.05–0.8 mg cm^−2^ and an I/C ratio of 0.29–1.67. The data collected allow for full understanding of the loss mechanisms for different I/C ratios, dependent on the platinum loadings and Pt/C ratio, and can ultimately be used for further analysis of gradient catalyst layers and as a data base for catalyst layer modeling and optimization. 

## 2. Experimental

### 2.1. Production of Catalyst Coated Membranes

A homogeneous suspension composed of platinum on carbon (40 wt.% Pt/C and 60 wt% Pt/C), Aquivion^®^ (D79-25BS, liquid dispersion, 25% in water, PFSA eq. wt. 790 g.mol^−1^ SO_3_H, stabilized CF_3_ polymer chain ends, Sigma-Aldrich Chemie GmbH, Schnelldorf, Germany) and a mixture of organic solvents (50 Vol.% ethylene glycol, 50 Vol.% propylene glycol methyl ether) was prepared. The suspension was homogenized by stirring. Suspensions with varying ionomer content were prepared with 15, 20, 25, 30, 35, 40, 45 and 50 wt.% ionomer content in dry layers. For both types of catalyst, this resulted in an I/C ratio in the range of 0.29–1.67. Catalyst layers with various platinum loadings were prepared by screen printing several layers on top of each other with subsequent drying at 110 °C. All catalyst layers were transferred onto a Gore membrane M735.18 containing an anode catalyst layer with a Pt loading of 0.05 mg cm^−2^. The transfer was carried out at a compression of 5 MPa (referred to the printed catalyst area of 20 cm^2^) and 180 °C for 15 min, producing CCMs with an active area of 12 cm^2^. As a gas diffusion layer on both the cathode and anode sides, a H23C9 GDL from Freudenberg was used.

The break-in procedure was accomplished by operating the cell at 80 °C and fully humidified gases (H_2_/Air at anode/cathode). The cell was operated for 1 h at 1.5 A/cm^2^ before it was cycled for 4 hours between open circuit voltage (OCV) (10 s), 0.6 V (60 s) and 0.4 V (60 s).

### 2.2. In situ Analysis

In this study, the different transport properties and overall performance of catalyst layers, with varying ionomer content, catalyst loading and 2 types of catalyst, were studied in situ using several characterization techniques. The CCMs were assembled in a Baltic FuelCells quickConnect^®^ test cell “high amp” (in Schwerin, Germany) having straight channels and an active area of 3 × 4 cm^2^. The cell was compressed with 5 bara (absolute pressure), corresponding to a compression pressure of 0.8 MPa on the active cell area. All experiments were run at a cell temperature of 80 °C and with various humidification levels using bubbler humidifiers. 

In this work, the catalyst layers were characterized using the following measurement protocol:

1. Polarization curves—the polarization curves were recorded at 80 °C cell temperature with fully humidified gases on the anode and cathode (i.e., 80 °C dew point) and an operating pressure of 2.0 bara. A constant flow rate was used with 2.0 nL/min H_2_ (norm liter per minute) on the anode and 5.0 nL/min air on the cathode. The polarization curves were recorded in potentiostatic mode from 0.2 V to OCV with 0.05 V increments from 0.20–0.75 V and 0.02 V increments from 0.78 - OCV. The holding times at each potential were potential dependent (U > 0.90 V: 30 s, 0.70 V < U ≤ 0.90 V: 60 s, and U ≤ 0.70 V: 5 min). A recovery procedure was also followed, where PtO was reduced at a voltage of 0.40 V for 5 s for load points above 0.75.
2. Cyclic voltammetry—the cyclic voltammetry was run between 0.05 and 0.60 V at 100 mV/s with a total of 5 cycles. For the ECSA calculation, an average of the last three cycles is taken. The operating temperature was 80 °C with fully humidified gases and at 1.0 bara.
3. Humidity Sweeps—the humidity sweeps were measured at 80 °C cell temperature and 1.5 bara pressure. The cell was operated at a constant load of 1 A/cm^2^ with fully humidified gases on the anode while the relative gas humidity on the cathode was varied between 20 % and 120 %. The voltage change was measured after each humidity step was conditioned for 7 min.

## 3. Results and Discussion

### 3.1. Ex Situ Analysis

**Figure 1:**
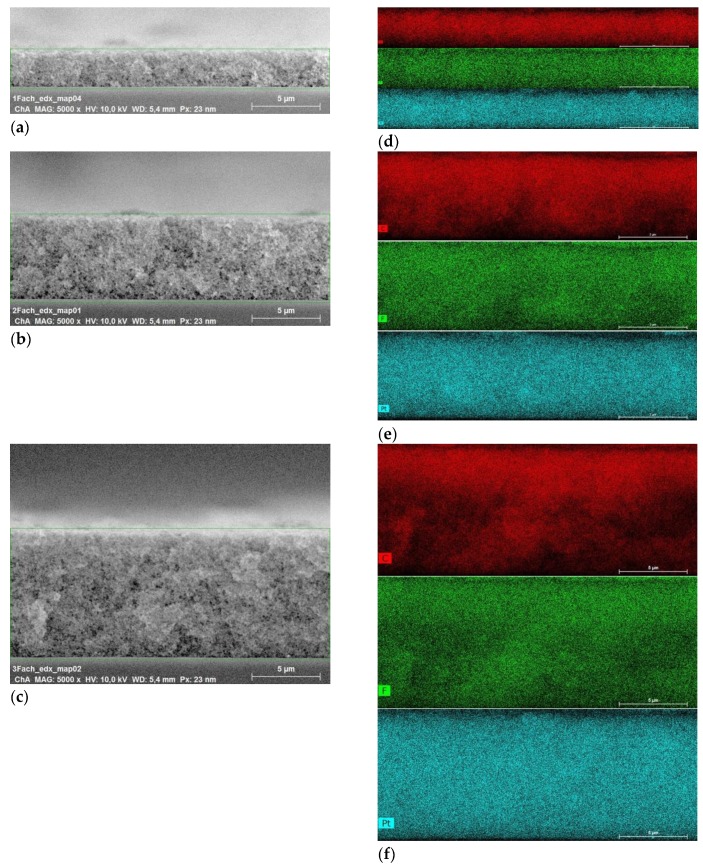
SEM images of three catalyst layers with an ionomer content of 30 wt.% and a platinum loading of (**a**) 0.08 mg cm^−2^ (1 Layer), (**b**) 0.180 mg cm^−2^ (2 Layers) and (**c**) 0.271 mg cm^−2^ (3 Layers) with the corresponding carbon, fluorine and platinum distributions given in (**d**) 1 layer, (**e**) 2 layers and (**f**) 3 layers: red corresponds to carbon, green corresponds to fluorine and turquoise to platinum.

In order to obtain the desired Pt loading, the catalyst layers were produced by printing various layers on top of each other. It is, hence, important to understand the interaction between these layers and how such a production step affects the interface between them. To do so, SEM images of three catalyst layers were taken and analyzed, as shown in Figure 1. As it can be seen from Figure 1a–c, there are no obvious interfaces visible that would indicate that interfacial effects might influence the performance of the layers. In Figure 1d–f, EDX distributions of carbon, fluorine and platinum are shown, where it can be seen that the ionomer, catalyst and support are homogeneously distributed throughout the different layers, regardless of the number of layers used for the production of the catalyst layer.

**Figure 2:**
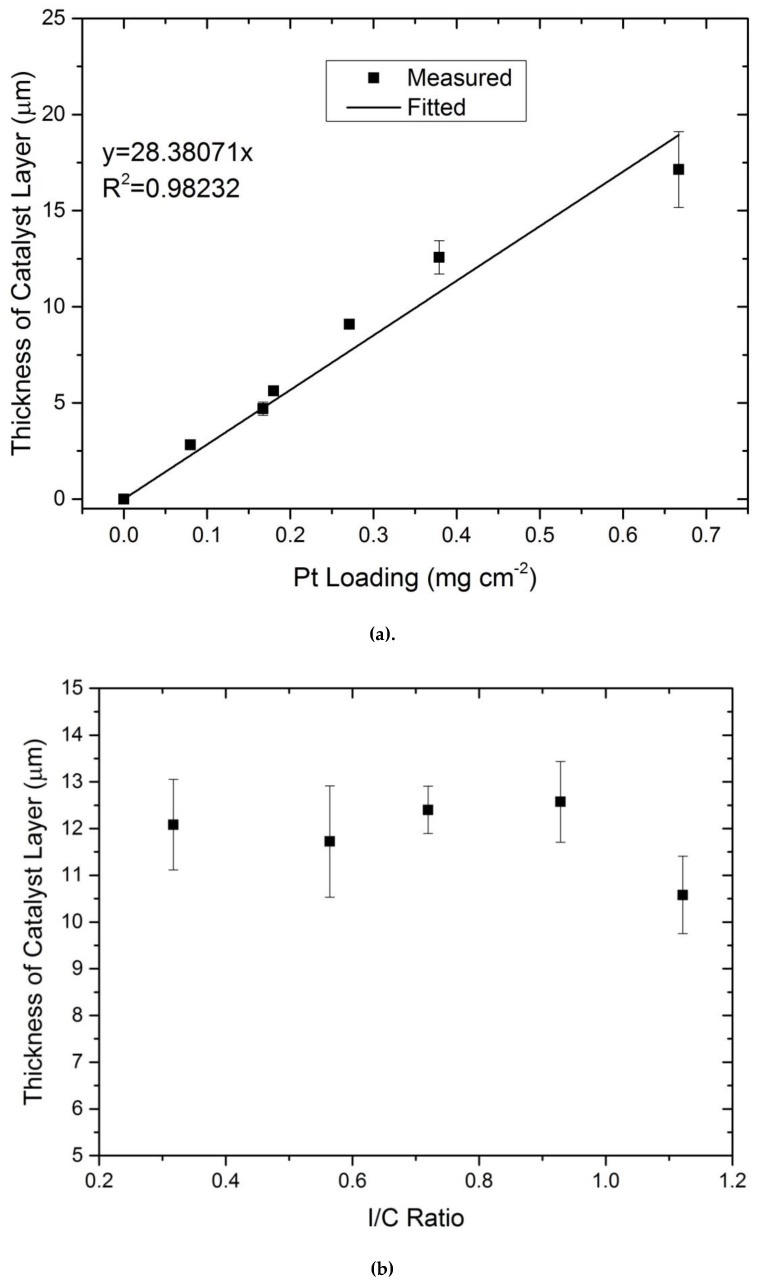
Dependence of catalyst layer thickness for the 40 wt% catalyst. (**a**) Measured thickness with an ionomer content of 35 wt.% dependent on the platinum loading, (**b**) effect of ionomer content on the thickness of the catalyst layer with Pt loading of 0.40 mg/cm^2^.

The dependency of the thickness on Pt loading and ionomer content was measured using SEM. In Figure 2, the thickness change with Pt loading for catalyst layers produced with 35 wt.% ionomer is given. As it can be seen, the thickness changes linearly with the change of Pt loading, while the ionomer content has no effect on the overall thickness of the catalyst layer. This implies constant porosity throughout the whole thickness of the produced electrodes.

### 3.2. Reproducibility of Produced CCMs

**Figure 3:**
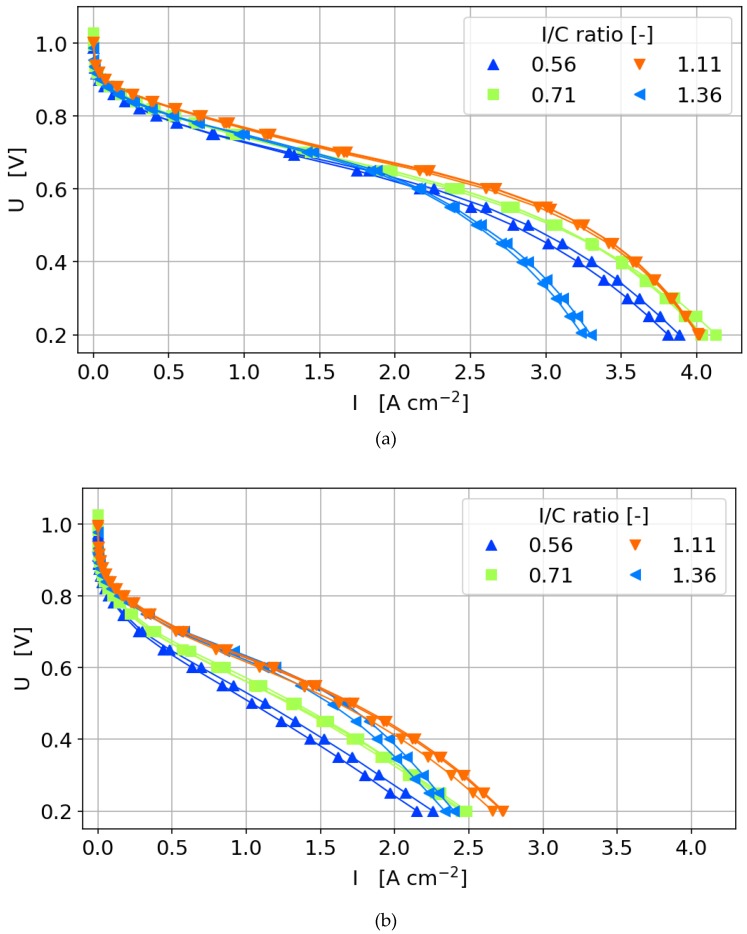

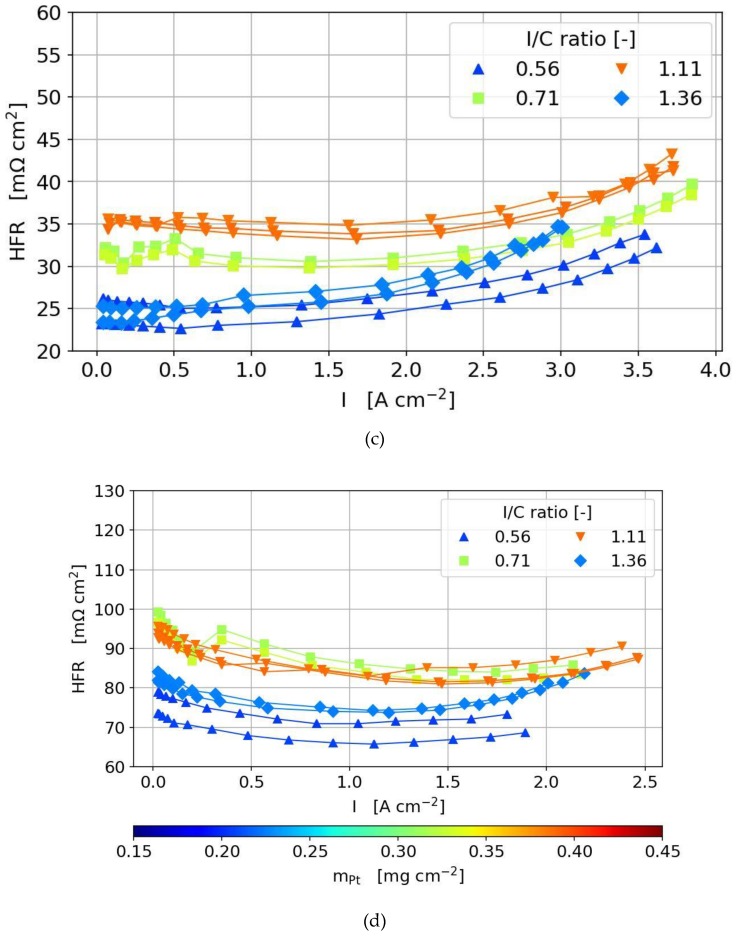
Polarization curves with varying Pt loading and I/C ratio under (**a**) wet conditions, (**b**) dry conditions to illustrate the reproducibility of the production method and characterization technique with their corresponding high frequency resistance under (**c**) wet conditions and (**d**) dry conditions.

Prior to investigating the effects of the catalyst layer ingredients on the performance of the cell, it is important to establish the reproducibility of the production and characterization method of the CCMs. In Figure 3a,b, the polarization curves under the wet and dry conditions of various CCMs with different Pt loadings and I/C ratios are given. Figure 3c,d shows the corresponding high frequency resistance (HFR) measurements during the polarization curves. As it can be seen, the production method used in this study results in reproducible catalyst layers and characterization results. 

### 3.3. In situ Analysis

The produced catalyst layers were characterized using the different in situ characterization techniques discussed earlier. In this work, we provide a full data set to understand the effects of various catalyst layer compositions. 

#### 3.3.1. Cyclic Voltammetry

**Figure 4:**
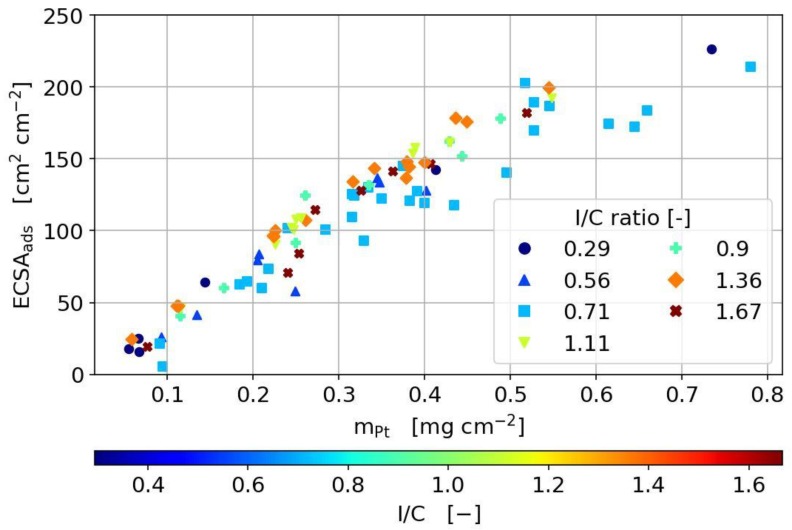
**Electrochemical active surface area**, ECSA, of the different catalyst coated membranes, CCMs, produced with 40wt% Pt/C, dependent on platinum loading and ionomer content.

The cyclic voltammetry was used in this study in order to estimate the electrochemical surface area (ECSA) for all produced catalyst layers. As shown in Figure 4, the ECSA is plotted against the Pt loading for various I/C ratios. The Pt loading was determined by weighing the catalyst layers after drying on the decal foil with the assumption that all solvent evaporates completely during drying. The ECSA was obtained by integrating the hydrogen adsorption current in the cyclic voltammograms until a fixed potential limit of 90 mV. 

As it can be seen, a linear dependency of the ECSA on Pt loading is measured for all I/C ratios. This linear dependency implies, (i) the calculation of the Pt loading is adequate at each respective ionomer content and (ii) proper electrical and protonic contact is established for each CL investigated, regardless of how many layers are printed to obtain the desired Pt loading.

#### 3.3.2. Polarization Curves

As mentioned earlier, the performance of the produced CCMs was characterized using two polarization curves, a dry curve (dew point 59 °C) and a fully humidified curve (dew point 80 °C). Figure 3a shows the fully humidified polarization curves and HFR data obtained with an I/C ratio of 0.56–1.36 with varying platinum loadings. The HFR is in a low and narrow range between 25 and 35 mOhm cm^2^, showing that the used production and characterization procedure is stable, and influences by variations of membrane properties can be neglected. This was also found for the other ionomer contents analyzed (data not shown). The influence of the platinum on the current production varies within the different regimes of the polarization curve. In the activation region, there is a clear trend with higher performance for higher catalyst loadings, whereas at higher currents the highest performance is not reached with the highest catalyst loading.

**Figure 5:**
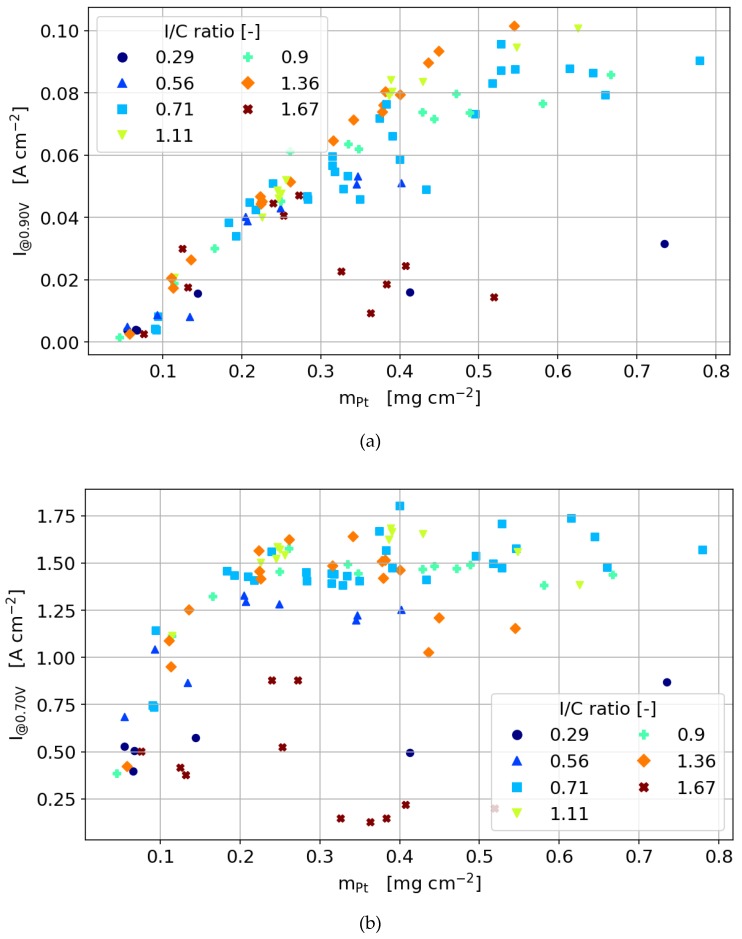

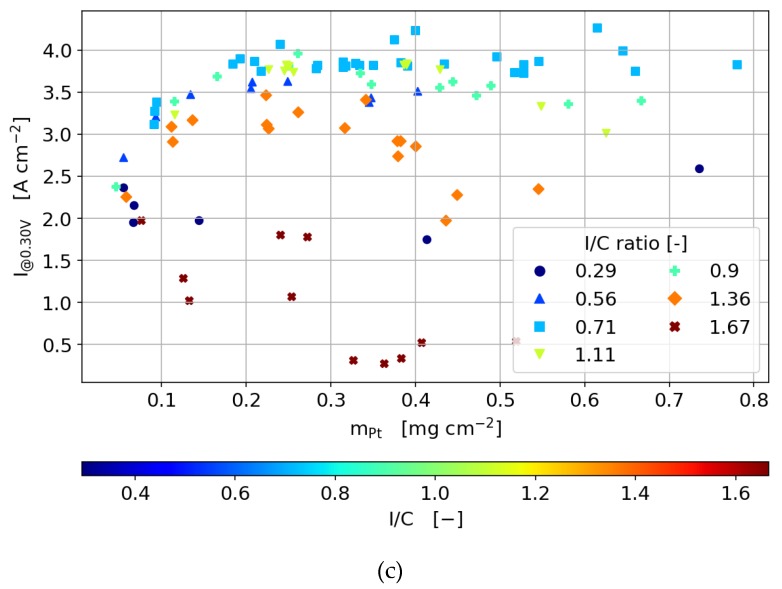
The current density dependency on the Pt loading and I/C ratio at cell voltages of (**a**) 900 mV, (**b**) 700 mV and (**c**) 300 mV for a catalyst of 40 wt% Pt/C.

**Figure 6:**
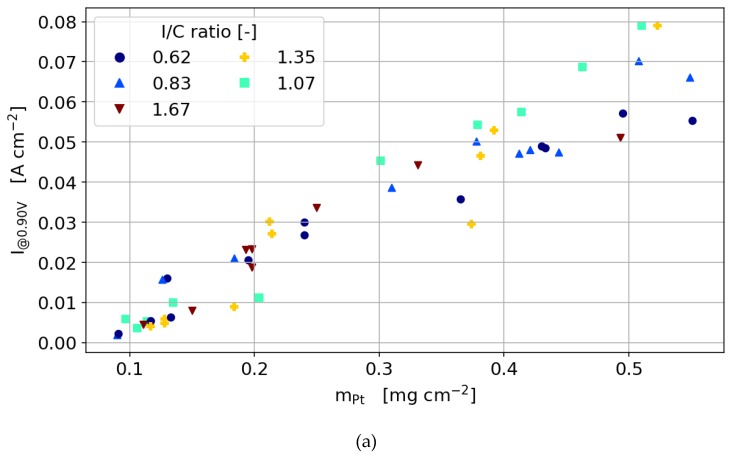

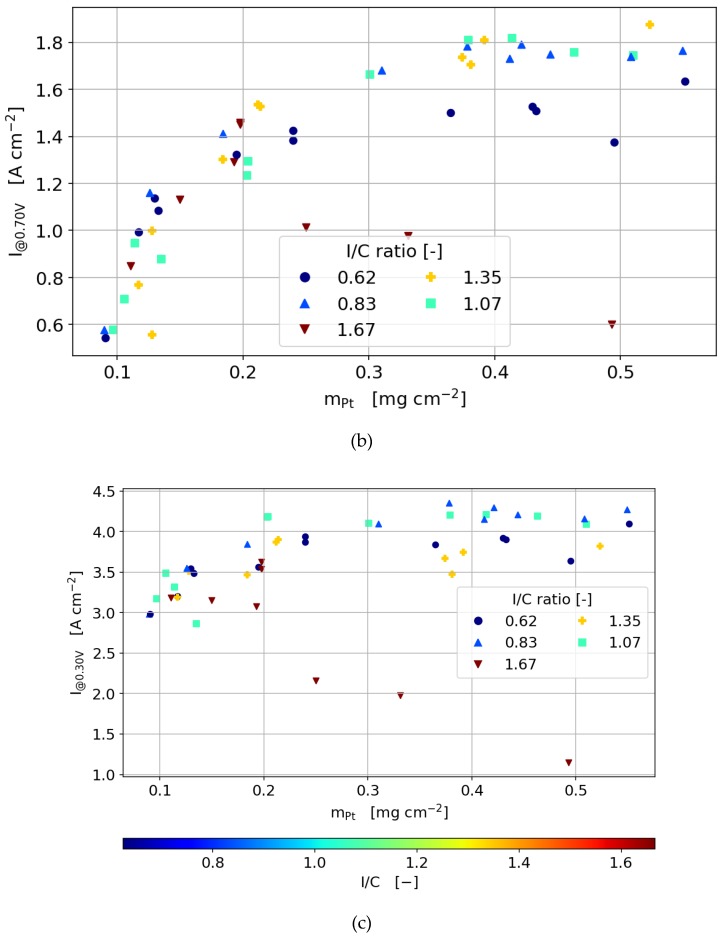
The current density dependency on the Pt loading and ionomer content at cell voltages of (**a**) 900 mV, (**b**) 700 mV and (**c**) 300 mV for a catalyst of 60 wt% Pt/C.

For better visualization of the performance of all 80+ cells discussed in this work, we chose to focus on three regions in the polarization curve as shown in Figure 5 and Figure 6. The three discrete points are extracted from the fully humidified polarization curves: From the activation region (900 mV, Figure 5a), the intermediate current density region (700 mV, Figure 5b) and the mass transport dominated region (300 mV, Figure 5c). Figure 5 shows the current density dependent on I/C ratio (indicated by color and marker type) and platinum loading for the 40 wt% catalyst. Figure 6 shows the same dependencies for the 60 wt% catalyst. In the following section, the general dependencies of both of the 40% catalysts will be discussed first, while the differences to the 60 wt% catalyst will be discussed later in this section.

#### 3.3.3. 40 wt% Catalyst

In the activation region in Figure 5a, a linear trend is measured for the dependency of the current density on the Pt loading, implying that the oxygen and proton transport effect on the performance is negligible due to the low reaction rates. However, for the catalyst layers with the highest and lowest ionomer content, the voltage losses due to proton transport and oxygen diffusion, respectively, are found to be high enough for a significantly lower current density for thick catalyst layers. The linear dependency measured for I/C ratios of 0.62–1.36 confirms the previous finding that, independent of the number of layers printed on top of each other, the whole catalyst layer thickness is electrochemically active and the layers are connected to both the protonic and electronic phases.

At platinum loadings higher than 0.3 mg cm^−2^, minor differences between the ionomer contents emerge, with an optimum I/C ratio of about 0.71–1.36. The fact that the optimum is found at a relatively high ionomer content indicates that high proton conductivity is more important than oxygen transport at the very low current densities in the activation region. For lower platinum loadings, the performance is rather independent of ionomer content.

At intermediate and high current densities (Figure 5b,c), mass transport and ohmic losses throughout the catalyst layer thickness become more pronounced due to the higher proton flux and oxygen consumption. Since the catalyst layer thickness varies proportionally with the platinum loading, both losses become more relevant for higher platinum loadings. At the higher current densities at 700 mV in Figure 5b and 300 mV in Figure 5c, the current density increases with platinum loading only up to a threshold value. At 700 mV, this threshold is around 0.3 mg cm^−2^ for all ionomer contents. At high ionomer loadings, even a decreasing performance can be observed for increasing platinum loading. Above 0.3 mg cm^−2^, adding further electrochemically active material does not result in a performance increase anymore because diffusive losses become dominant over the performance increase by the increasing the electrochemical active area.

For the low ionomer contents, adding additional material simply results in more inactive material and the current density remains unchanged for higher platinum loadings. This is only possible since for the low ionomer contents, the oxygen diffusion is still high enough to supply the catalyst close to the membrane. Otherwise, adding more material would decrease the cell current by higher diffusion losses due to the increase in the layer’s thickness with increased Pt loading.

For I/C ratios higher than 1.07, higher platinum loading results in a performance decrease and a more pronounced platinum loading optimum. Upon the increase in ionomer content over the optimum, the diffusion through the thickness of the catalyst layer seems to limit the cell performance. With increasing catalyst layer thicknesses, (or Pt loading), the oxygen diffusion to the mainly active regions close to the membrane is hindered, which results in moving the reaction zone away from the membrane towards the interface to the GDL. The fact that the current is even decreasing with increasing the platinum loading indicates that despite the high ionomer contents, the proton conductivity is not high enough to enable a proton transport to the reaction zone close to the GDL without significant losses. Increasing the thickness with higher platinum loading, therefore results in ohmic losses that overcompensate the effects by the higher platinum loading.

In Figure 5c, the cell current at 300 mV also shows a distinct optimum for the platinum loading that is also dependent on the ionomer content. Here, differences between the ionomer contents are more pronounced with the optimum at 300 mV around 0.2 mg Pt cm^−2^ compared to 0.3 mg Pt cm^−2^ at 700 mV. 

In the fuel cell literature, the discussion on whether the oxygen diffusion to the active sites in the catalyst layer is limited by diffusion through the ionomer film that covers the catalyst particles or by the through-plane diffusion in the pore space of the catalyst layer, is ongoing [21]. Analyzing the findings in the previous section, the results strongly support the hypothesis that the losses are dominated by the through-plane diffusion rather than the film diffusion. Adding thickness to the layer results in decreasing performance for all layers, especially for the higher ionomer contents. This would not be the case if thin-film diffusion would be mainly limiting. If the proton conductivity throughout the layer would prevent the performance to increase, this effect would be more pronounced for lower rather than higher ionomer loadings. 

Another indication that limitation by through-plane diffusion rather than its thin-film counterpart is observed through the analysis of catalyst layers with low ionomer contents at low layer thicknesses (low Pt loading). The performance difference between the different ionomer loadings seems to diminish below catalyst loadings smaller than 0.2 mg_Pt_ cm^−2^, resulting in a performance that is independent of ionomer loading for very thin catalyst layers. If the thin film diffusion would limit the cell performance, the differences between ionomer contents would be independent of catalyst layer thickness and also be present for the thin catalyst layers at low platinum loadings. 

#### 3.3.4. Humidity Sweeps

**Figure 7:**
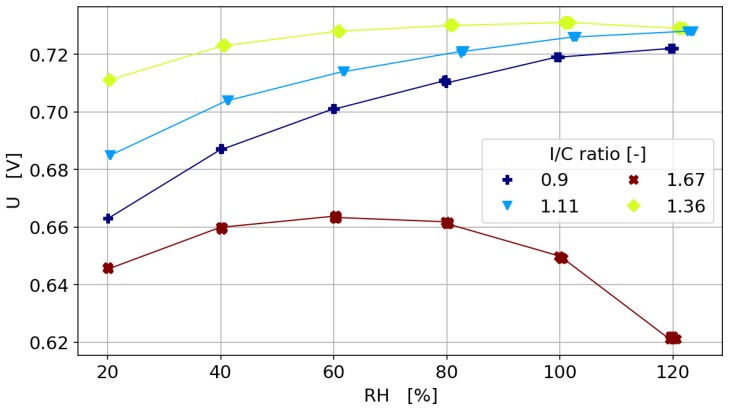
Relative humidity sweeps dependent on I/C ratio for Pt/C 40wt.% and a Pt loading of 0.25 mg cm^−2^ at a current density of 1.0 A cm^−2^.

**Figure 8:**
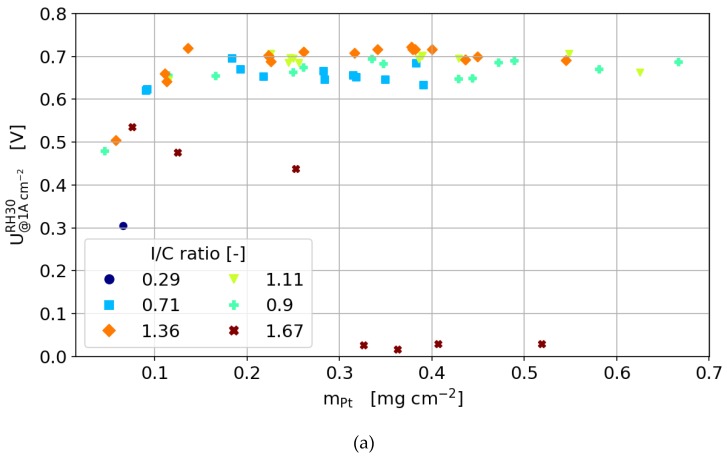

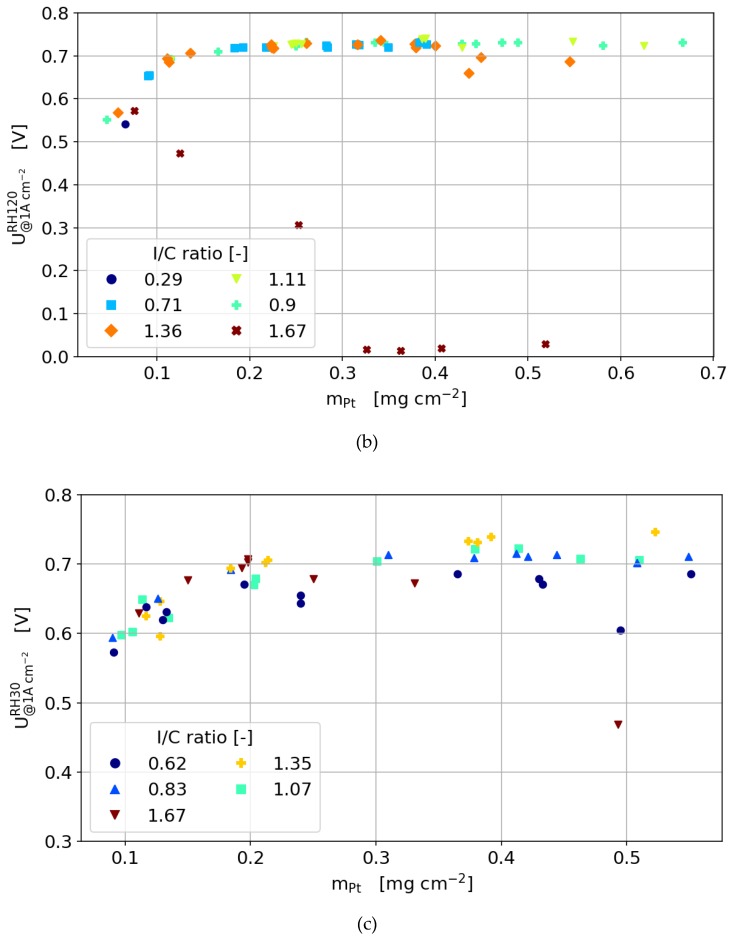

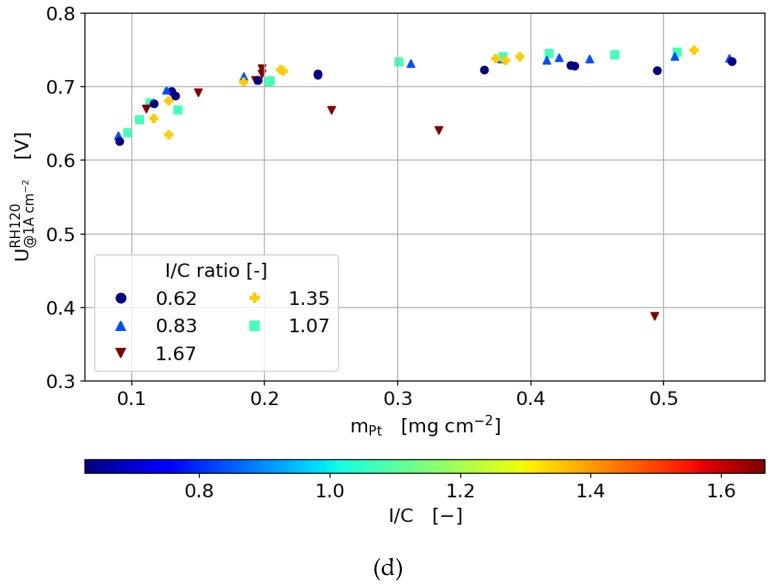
The cell voltage dependent on Pt loading and I/C ratio for a relative humidity of (**a**) 30% and (**b**) 120% for Pt/C of 40wt.% and a relative humidity of (**c**) 30% and (**d**) 120% for Pt/C of 60wt.%. All humidity sweeps are carried out at a current density of 1.0 A cm^−2^.

In order to investigate the effect of Pt loading and ionomer content on the overall water transport within the cell, relative humidity sweeps were carried out at a current density of 1.0 A cm^−2^. Figure 7 shows the humidity sweeps dependent on the I/C ratio for a Pt loading of 0.25 mg cm^−2^. It is obvious that the catalyst layers with low ionomer content suffer more from drying at low humidification than the catalyst layers with high ionomer content. To investigate this further, the cell voltage at 30% RH and 120% RH, dependent on catalyst loading, is plotted in Figure 8. For the dry conditions, the optimum I/C ratio is found to be at 1.36, while the optimum I/C ratio is found to be in the range of 0.62–1.36 for the wet conditions. Further, it can be seen that an ionomer content above 1.36 might result in flooding of the cell at high humidity levels.

#### 3.3.5. 60 wt % Catalyst

The general dependencies for the optimum ionomer content of the 60 wt% catalyst in terms of ionomer to carbon ratio is the same as for the 40 wt% catalyst. As found by other groups before, the amount of ionomer needed for an optimum compromise between oxygen diffusion and proton transport depends strongly on the carbon surface rather than on the amount of platinum in the catalyst layer. This results in a significantly lower weight percentage of ionomer needed for the 60 wt% catalyst than for the 40 wt% catalyst at the same platinum loading. This is highlighted in Figure 8c,d, where it can be seen that the cells are more tolerant to low humidity. 

When considering the change in performance with catalyst layer thickness (or platinum loading), the optimum performance of the 60 wt% catalyst is not as pronounced as that of the 40 wt % catalyst. This obviously results from the thickness difference between the two catalyst types. For the same platinum loading, the 60 wt% catalysts are about 33% thinner than the 40 wt% catalyst since mostly the amount of carbon defines the layer thickness. For higher platinum loadings, the diffusion limitation by the additional thickness is not as pronounced as for the 40 wt% catalyst. 

This supports the conclusion of the previous section, where the through-plane diffusion was found to be the main source for the limitations at higher platinum and ionomer loadings. Since the maximum in power output is defined by the interaction between through-plane diffusion and proton conduction, this optimum is shifted towards higher platinum loadings for the thinner catalyst layers with the 60 wt% catalyst. At 300 mV cell voltage, the optimum performance is therefore reached at around 0.25 mg cm^−2^ platinum loading for the 40 wt % catalyst and at around 0.3 mg cm^−2^ for the 60 wt % catalyst.

## 4. Conclusions

A full parametric study of various catalyst layer compositions containing the variation of platinum loading, ionomer content and two types of catalyst material (40 and 60 wt.% Pt/C) was conducted. The following conclusions can be drawn from the work presented in this manuscript.

1. Electrical and protonic contact is established regardless of the printing of layers on topic of each other. This conclusion was established based on SEM images that illustrated a homogeneous distribution of the Pt, C and F. The linear dependency of the ECSA on Pt loading for various I/C ratios without any discontinuity in the slope once again proves the establishment of the electrical and protonic contact between all layers.
2. The optimum ionomer content is not only dependent on the catalyst layer thickness, but also on the load and gas humidity.
3. In the activation region, regardless of which type of catalyst is used, the dependency of the current density on Pt loading is linear as long as the ionomer content is not too low to hinder proton conductivity and is not too high to hinder oxygen diffusion significantly.
4. In the ohmic and mass transport region of the polarization curve, increasing the ionomer content to a value higher than the optimum results in a decrease in current density with higher Pt loading. The diffusion losses outweigh the improvement by the increasing catalytically active area.
5. At high Pt loading and ionomer content, through-plane diffusion losses become more limiting than the proton conductivity.
6. General trends are the same for 40 and 60 wt.% Pt/C catalysts, but the dependency on ionomer content is lower due to reduced catalyst layer thickness.

The impact of this work is in the comprehensive data that it provides. The plan is to use this data set as a basis for our ongoing efforts to investigate the effect of catalyst layer structuring, specifically graded catalyst layers, on its performance. This investigation will be carried out by additionally measuring limiting current density and impedance spectroscopy, and will be further enhanced by accelerated stress tests to understand the dependencies of durability and stability on the structuring of the layer.
